# 7-(2,2-Dimethylpropanamido)-2-methyl-1,8-naphthyridin-1-ium chloride monohydrate

**DOI:** 10.1107/S1600536808042955

**Published:** 2009-01-17

**Authors:** Hoong-Kun Fun, Reza Kia, Nirmal Kumar Das, Debabrata Sen, Shyamaprosad Goswami

**Affiliations:** a X-ray Crystallography Unit, School of Physics, Universiti Sains Malaysia, 11800 USM, Penang, Malaysia; b Department of Chemistry, Bengal Engineering and Science University, Shibpur, Howrah 711 103, India

## Abstract

The asymmetric unit of the title compound, C_14_H_18_N_3_O^+^·Cl^−^·H_2_O, comprises a substituted amido–naphthyridine cation, a chloride anion and a water mol­ecule of crystallization. Intra­molecular C—H⋯O hydrogen bonds generate six-membered rings, producing an *S*(6) ring motif. The amido group is twisted from the naphthyridine ring, making a dihedral angle of 17.65 (7)°. The crystal structure is stabilized by inter­molecular N—H⋯O, N—H⋯Cl, O—H⋯Cl (× 2), and C—H⋯O (× 2) hydrogen bonds. These inter­actions linked neighbouring mol­ecules into chains along the *a* and *b* axes of the crystal, thus forming mol­ecular sheets parallel to the (001) plane.

## Related literature

For details of hydrogen-bond motifs, see: Bernstein *et al.* (1995 ▶). For biological activity and mol­ecular recognition, see: Goswami *et al.* (2005 ▶); Carmen *et al.* (2004 ▶); Goswami & Mukherjee (1997 ▶); Yu *et al.* (2008 ▶).
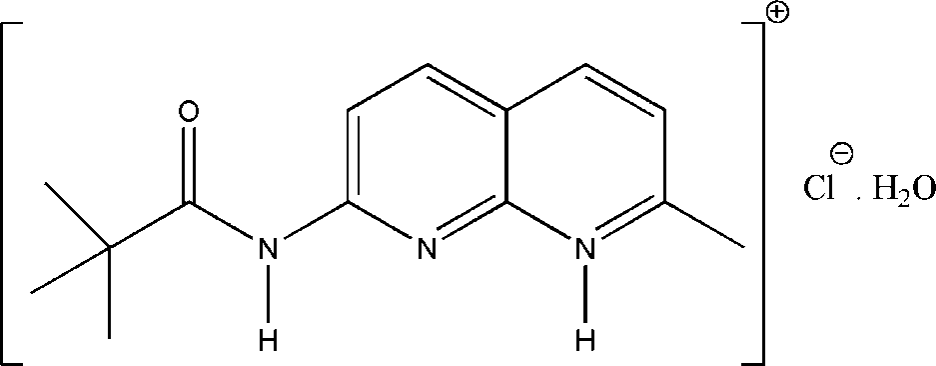


## Experimental

### 

#### Crystal data

C_14_H_18_N_3_O^+^·Cl^−^·H_2_O
*M* *_r_* = 297.78
Orthorhombic, 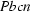

*a* = 19.0092 (5) Å
*b* = 9.0077 (2) Å
*c* = 17.7294 (5) Å
*V* = 3035.79 (14) Å^3^
*Z* = 8
Mo *K*α radiation
μ = 0.26 mm^−1^
*T* = 100.0 (1) K
0.41 × 0.29 × 0.19 mm

#### Data collection

Bruker SMART APEXII CCD area-detector diffractometer
Absorption correction: multi-scan (**SADABS**; Bruker, 2005 ▶) *T*_min_ = 0.902, *T*_max_ = 0.954
19927 measured reflections
4489 independent reflections
3470 reflections with *I* > 2σ(*I*)
*R*_int_ = 0.037

#### Refinement

*R*[*F* ^2^ > 2σ(*F* ^2^)] = 0.040
*wR*(*F* ^2^) = 0.102
*S* = 1.07
4489 reflections
198 parameters
H atoms treated by a mixture of independent and constrained refinement
Δρ_max_ = 0.36 e Å^−3^
Δρ_min_ = −0.27 e Å^−3^

### 

Data collection: *APEX2* (Bruker, 2005 ▶); cell refinement: *SAINT* (Bruker, 2005 ▶)’; data reduction: *SAINT*; program(s) used to solve structure: *SHELXTL* (Sheldrick, 2008 ▶); program(s) used to refine structure: *SHELXTL*; molecular graphics: *SHELXTL*; software used to prepare material for publication: *SHELXTL* and *PLATON* (Spek, 2003 ▶).

## Supplementary Material

Crystal structure: contains datablocks global, I. DOI: 10.1107/S1600536808042955/ng2528sup1.cif

Structure factors: contains datablocks I. DOI: 10.1107/S1600536808042955/ng2528Isup2.hkl

Additional supplementary materials:  crystallographic information; 3D view; checkCIF report

## Figures and Tables

**Table 1:** Hydrogen-bond geometry (Å, °)

*D*—H⋯*A*	*D*—H	H⋯*A*	*D*⋯*A*	*D*—H⋯*A*
N1—H1*N*1⋯O1*W*	0.833 (18)	2.041 (17)	2.8633 (16)	169.1 (16)
N3—H1*N*3⋯Cl1	0.877 (18)	2.213 (18)	3.0870 (11)	175.2 (16)
O1*W*—H1*W*1⋯Cl1^i^	0.891 (19)	2.219 (19)	3.1091 (12)	176.5 (18)
O1*W*—H2*W*1⋯Cl1	0.85 (2)	2.61 (2)	3.3960 (12)	155.3 (16)
C7—H7*A*⋯O1	0.93	2.27	2.8298 (17)	118
C11—H11*A*⋯O1^ii^	0.96	2.54	3.3742 (18)	145
C13—H13*A*⋯O1*W*	0.96	2.60	3.4997 (18)	157

Symmetry codes: (i) 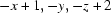; (ii) 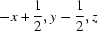.

**Fig. 1.**
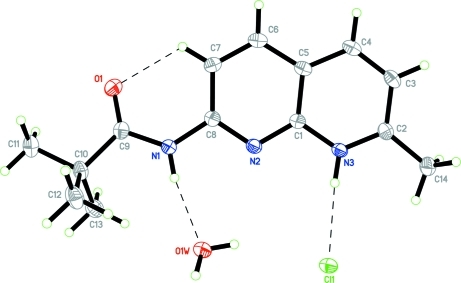
The molecular structure of (I), showing 50% probability displacement ellipsoids and the atomic numbering. Dashed line show intramolecular hydrogen bond.

**Fig. 2.**
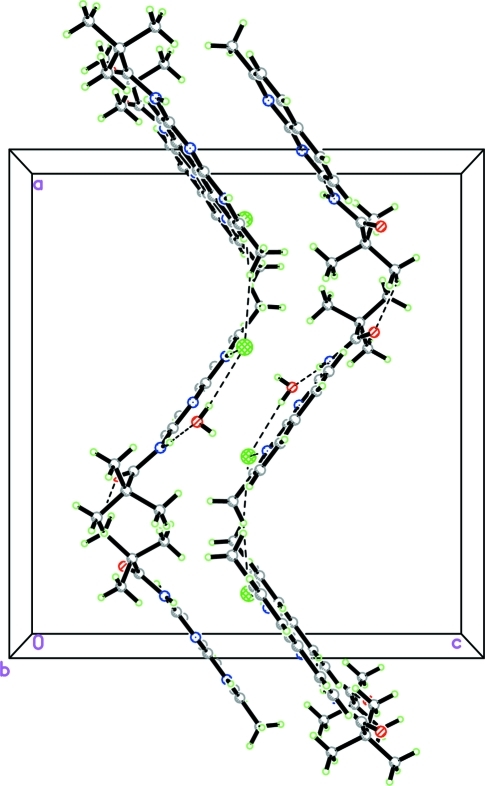
The crystal packing for (I), viewed down the b-axis showing linking of molecules along the a-axis. Intermolecular interactions are drawn as dashed lines.

## supplementary crystallographic information

### Comment

Naphthyridine or naphthyridone systems are of great importance due to their broad spectrum of biological activities. Substituted 1,8-naphthyridine compounds are used as antihypertensives, antiarrhythmics, herbicide safeners and also as immunostimulants (Goswami *et al.,* 2005). Naphthyridine molecules also have interesting crystal structures (Carmen *et al.,* 2004) and are used in molecular recognition chemistry (Goswami *et al.,* 2005; Yu *et al.,* 2008).

In the title compound (I), Fig. 1, intramolecular C—H···O hydrogen bond generates six-membered ring, producing *S(6)* ring motif (Bernstein *et al.,* 1995). The chloride anion and water molecule are mediated to link neighbouring molecules together through hydrogen bonds. The amido group is twisted from the naphthyridine ring making a dihedral angle of 17.65 (7)°. The crystal structure is stabilized by intermolecular N—H···O, N—H···Cl, O—H···Cl(*x* 2), and C—H···O (*x* 2) hydrogen bonds. These interactions linked neighbouring molecules together as chains along the *a* and *b* axes of the crystal, thus forming 2-D molecular sheets parallel to the (001) plane.

### Experimental

In a round bottom flask, 7-methyl-[1,8]naphthyridin-2-ylamine (100 mg, 0.63 mmol) and triethyl amine (0.1 mL) were stirred in dry dichloromethane (1 mL) under nitrogen at 0 ^°^ C. Pivaloyl chloride (0.116 mL, 0.95 mmol) was then added dropwise. After 1 h, the solvent was removed and the residue was neutralized with saturated NaHCO_3_ and fresh dichloromethane was added. The organic part was collected and removed under reduced pressure. The crude product was then purified by column chromatography using ethylacetate and petroleum ether (1:1) which offered the entitled compound as an off-white crystalline solid (82 mg, 53%), m.p. 66-68 ^°^ C.

### Refinement

Hydrogen atoms of the water molecule and N-bound H atoms were located from the difference Fourier map and refined freely, see Table 1. The rest of the hydrogen atoms were positioned geometrically and constrained to refine with the parent atoms with U_iso_ (H) = 1.2 or 1.5 U_eq_ (C). A rotating group model applied for the methyl group bound to the naphthyridine ring.

### Crystal data

**Table:**

C_14_H_18_N_3_O^+^·Cl^−^·H_2_O	*F*(000) = 1264
*M**_r_* = 297.78	*D*_x_ = 1.303 Mg m^−^^3^
Orthorhombic, *P**b**c**n*	Mo *K*α radiation, λ = 0.71073 Å
Hall symbol: -P 2n 2ab	Cell parameters from 5042 reflections
*a* = 19.0092 (5) Å	θ = 2.3–29.9°
*b* = 9.0077 (2) Å	µ = 0.26 mm^−^^1^
*c* = 17.7294 (5) Å	*T* = 100 K
*V* = 3035.79 (14) Å^3^	Block, colourless
*Z* = 8	0.41 × 0.29 × 0.19 mm

### Data collection

**Table:**

Bruker SMART APEXII CCD area-detector diffractometer	4489 independent reflections
Radiation source: fine-focus sealed tube	3470 reflections with *I* > 2σ(*I*)
graphite	*R*_int_ = 0.037
φ and ω scans	θ_max_ = 30.2°, θ_min_ = 2.1°
Absorption correction: multi-scan (*SADABS*; Bruker, 2005)	*h* = −26→23
*T*_min_ = 0.902, *T*_max_ = 0.954	*k* = −12→12
19927 measured reflections	*l* = −25→18

### Refinement

**Table:**

Refinement on *F*^2^	Primary atom site location: structure-invariant direct methods
Least-squares matrix: full	Secondary atom site location: difference Fourier map
*R*[*F*^2^ > 2σ(*F*^2^)] = 0.040	Hydrogen site location: inferred from neighbouring sites
*wR*(*F*^2^) = 0.102	H atoms treated by a mixture of independent and constrained refinement
*S* = 1.07	*w* = 1/[σ^2^(*F*_o_^2^) + (0.0453*P*)^2^ + 0.653*P*] where *P* = (*F*_o_^2^ + 2*F*_c_^2^)/3
4489 reflections	(Δ/σ)_max_ = 0.001
198 parameters	Δρ_max_ = 0.36 e Å^−^^3^
0 restraints	Δρ_min_ = −0.27 e Å^−^^3^

### Special details

**Table:**

Experimental. The low-temperature data was collected with the Oxford Cyrosystem Cobra low-temperature attachment.
Geometry. All esds (except the esd in the dihedral angle between two l.s. planes) are estimated using the full covariance matrix. The cell esds are taken into account individually in the estimation of esds in distances, angles and torsion angles; correlations between esds in cell parameters are only used when they are defined by crystal symmetry. An approximate (isotropic) treatment of cell esds is used for estimating esds involving l.s. planes.
Refinement. Refinement of F^2^ against ALL reflections. The weighted R-factor wR and goodness of fit S are based on F^2^, conventional R-factors R are based on F, with F set to zero for negative F^2^. The threshold expression of F^2^ > 2sigma(F^2^) is used only for calculating R-factors(gt) etc. and is not relevant to the choice of reflections for refinement. R-factors based on F^2^ are statistically about twice as large as those based on F, and R- factors based on ALL data will be even larger.

### Fractional atomic coordinates and isotropic or equivalent isotropic displacement parameters (Å^2^)

**Table:**

	*x*	*y*	*z*	*U*_iso_*/*U*_eq_	
Cl1	0.613003 (17)	0.14014 (4)	0.995585 (18)	0.02130 (9)	
O1	0.35100 (5)	0.43024 (11)	0.71527 (5)	0.0247 (2)	
N1	0.41076 (6)	0.32279 (13)	0.81210 (7)	0.0197 (2)	
N2	0.50292 (6)	0.40348 (12)	0.88207 (6)	0.0180 (2)	
N3	0.59795 (6)	0.47140 (12)	0.95397 (6)	0.0174 (2)	
C1	0.54282 (6)	0.51370 (14)	0.90903 (7)	0.0166 (3)	
C2	0.64346 (7)	0.56681 (15)	0.98399 (7)	0.0194 (3)	
C3	0.63409 (7)	0.71897 (15)	0.97088 (8)	0.0224 (3)	
H3A	0.6653	0.7870	0.9918	0.027*	
C4	0.57933 (7)	0.76783 (15)	0.92753 (8)	0.0218 (3)	
H4A	0.5730	0.8690	0.9196	0.026*	
C5	0.53258 (7)	0.66552 (14)	0.89490 (7)	0.0182 (3)	
C6	0.47437 (7)	0.70180 (15)	0.84855 (8)	0.0216 (3)	
H6A	0.4641	0.8006	0.8381	0.026*	
C7	0.43369 (7)	0.59217 (15)	0.81942 (8)	0.0209 (3)	
H7A	0.3958	0.6147	0.7883	0.025*	
C8	0.45006 (7)	0.44204 (14)	0.83733 (7)	0.0178 (3)	
C9	0.36274 (7)	0.32093 (15)	0.75354 (7)	0.0191 (3)	
C10	0.32593 (7)	0.17247 (15)	0.74131 (7)	0.0209 (3)	
C11	0.27199 (8)	0.19006 (18)	0.67779 (8)	0.0276 (3)	
H11A	0.2482	0.0973	0.6698	0.041*	
H11B	0.2956	0.2190	0.6323	0.041*	
H11C	0.2383	0.2648	0.6914	0.041*	
C12	0.28790 (8)	0.12662 (18)	0.81407 (8)	0.0288 (3)	
H12A	0.3216	0.1148	0.8539	0.043*	
H12B	0.2637	0.0344	0.8060	0.043*	
H12C	0.2545	0.2020	0.8278	0.043*	
C13	0.38030 (8)	0.05470 (16)	0.71945 (8)	0.0272 (3)	
H13A	0.4136	0.0431	0.7597	0.041*	
H13B	0.4044	0.0853	0.6745	0.041*	
H13C	0.3569	−0.0381	0.7105	0.041*	
C14	0.70189 (7)	0.50748 (17)	1.03071 (8)	0.0254 (3)	
H14A	0.6971	0.4017	1.0352	0.038*	
H14B	0.7003	0.5518	1.0799	0.038*	
H14C	0.7460	0.5305	1.0072	0.038*	
O1W	0.46432 (6)	0.07427 (12)	0.89481 (6)	0.0233 (2)	
H1N1	0.4218 (9)	0.244 (2)	0.8337 (9)	0.028 (4)*	
H1N3	0.6013 (9)	0.376 (2)	0.9631 (11)	0.037 (5)*	
H1W1	0.4421 (10)	0.016 (2)	0.9277 (11)	0.042 (5)*	
H2W1	0.4959 (11)	0.119 (2)	0.9197 (11)	0.048 (6)*	

### Atomic displacement parameters (Å^2^)

**Table:**

	*U*^11^	*U*^22^	*U*^33^	*U*^12^	*U*^13^	*U*^23^
Cl1	0.02288 (18)	0.01664 (16)	0.02439 (17)	−0.00058 (12)	0.00160 (12)	0.00273 (13)
O1	0.0238 (5)	0.0249 (5)	0.0254 (5)	0.0023 (4)	−0.0030 (4)	0.0028 (4)
N1	0.0191 (6)	0.0159 (6)	0.0241 (6)	−0.0016 (4)	−0.0036 (4)	0.0020 (5)
N2	0.0171 (5)	0.0146 (5)	0.0223 (5)	−0.0005 (4)	−0.0002 (4)	0.0004 (4)
N3	0.0182 (5)	0.0136 (5)	0.0204 (5)	−0.0008 (4)	0.0004 (4)	0.0003 (4)
C1	0.0163 (6)	0.0157 (6)	0.0177 (6)	0.0000 (5)	0.0027 (5)	0.0003 (5)
C2	0.0182 (6)	0.0200 (7)	0.0200 (6)	−0.0025 (5)	0.0013 (5)	−0.0020 (5)
C3	0.0232 (7)	0.0193 (7)	0.0247 (7)	−0.0064 (5)	0.0020 (5)	−0.0026 (6)
C4	0.0265 (7)	0.0146 (6)	0.0242 (7)	−0.0022 (5)	0.0039 (5)	−0.0005 (5)
C5	0.0211 (7)	0.0147 (6)	0.0187 (6)	−0.0002 (5)	0.0042 (5)	−0.0004 (5)
C6	0.0250 (7)	0.0159 (6)	0.0238 (6)	0.0027 (5)	0.0027 (5)	0.0015 (5)
C7	0.0211 (7)	0.0188 (6)	0.0228 (6)	0.0029 (5)	−0.0013 (5)	0.0018 (5)
C8	0.0163 (6)	0.0177 (6)	0.0193 (6)	0.0001 (5)	0.0022 (5)	−0.0001 (5)
C9	0.0146 (6)	0.0235 (7)	0.0191 (6)	0.0011 (5)	0.0033 (5)	−0.0016 (5)
C10	0.0187 (7)	0.0253 (7)	0.0187 (6)	−0.0052 (5)	0.0017 (5)	−0.0023 (5)
C11	0.0221 (7)	0.0360 (8)	0.0248 (7)	−0.0061 (6)	−0.0017 (6)	−0.0019 (6)
C12	0.0277 (8)	0.0362 (9)	0.0226 (7)	−0.0125 (7)	0.0037 (6)	−0.0010 (6)
C13	0.0301 (8)	0.0246 (7)	0.0270 (7)	−0.0018 (6)	0.0006 (6)	−0.0048 (6)
C14	0.0207 (7)	0.0254 (7)	0.0300 (7)	−0.0025 (6)	−0.0049 (6)	−0.0010 (6)
O1W	0.0259 (6)	0.0190 (5)	0.0251 (5)	−0.0034 (4)	−0.0011 (4)	0.0035 (4)

### Geometric parameters (Å, °)

**Table:**

O1—C9	1.2164 (16)	C7—H7A	0.9300
N1—C9	1.3824 (17)	C9—C10	1.5248 (19)
N1—C8	1.3827 (17)	C10—C13	1.531 (2)
N1—H1N1	0.834 (17)	C10—C11	1.5312 (19)
N2—C8	1.3266 (17)	C10—C12	1.5353 (19)
N2—C1	1.3377 (16)	C11—H11A	0.9600
N3—C2	1.3305 (17)	C11—H11B	0.9600
N3—C1	1.3705 (17)	C11—H11C	0.9600
N3—H1N3	0.881 (18)	C12—H12A	0.9600
C1—C5	1.4039 (18)	C12—H12B	0.9600
C2—C3	1.401 (2)	C12—H12C	0.9600
C2—C14	1.4851 (19)	C13—H13A	0.9600
C3—C4	1.367 (2)	C13—H13B	0.9600
C3—H3A	0.9300	C13—H13C	0.9600
C4—C5	1.4050 (19)	C14—H14A	0.9600
C4—H4A	0.9300	C14—H14B	0.9600
C5—C6	1.4163 (19)	C14—H14C	0.9600
C6—C7	1.3565 (19)	O1W—H1W1	0.89 (2)
C6—H6A	0.9300	O1W—H2W1	0.85 (2)
C7—C8	1.4236 (18)		
			
C9—N1—C8	127.52 (12)	N1—C9—C10	114.86 (12)
C9—N1—H1N1	120.1 (12)	C9—C10—C13	109.50 (11)
C8—N1—H1N1	112.1 (12)	C9—C10—C11	108.73 (12)
C8—N2—C1	116.67 (11)	C13—C10—C11	109.72 (11)
C2—N3—C1	123.38 (12)	C9—C10—C12	109.43 (11)
C2—N3—H1N3	120.8 (12)	C13—C10—C12	110.14 (12)
C1—N3—H1N3	115.8 (12)	C11—C10—C12	109.30 (11)
N2—C1—N3	115.78 (11)	C10—C11—H11A	109.5
N2—C1—C5	125.49 (12)	C10—C11—H11B	109.5
N3—C1—C5	118.73 (12)	H11A—C11—H11B	109.5
N3—C2—C3	118.86 (12)	C10—C11—H11C	109.5
N3—C2—C14	118.48 (12)	H11A—C11—H11C	109.5
C3—C2—C14	122.65 (13)	H11B—C11—H11C	109.5
C4—C3—C2	120.34 (13)	C10—C12—H12A	109.5
C4—C3—H3A	119.8	C10—C12—H12B	109.5
C2—C3—H3A	119.8	H12A—C12—H12B	109.5
C3—C4—C5	120.14 (13)	C10—C12—H12C	109.5
C3—C4—H4A	119.9	H12A—C12—H12C	109.5
C5—C4—H4A	119.9	H12B—C12—H12C	109.5
C1—C5—C4	118.54 (12)	C10—C13—H13A	109.5
C1—C5—C6	115.89 (12)	C10—C13—H13B	109.5
C4—C5—C6	125.57 (12)	H13A—C13—H13B	109.5
C7—C6—C5	119.89 (13)	C10—C13—H13C	109.5
C7—C6—H6A	120.1	H13A—C13—H13C	109.5
C5—C6—H6A	120.1	H13B—C13—H13C	109.5
C6—C7—C8	118.81 (13)	C2—C14—H14A	109.5
C6—C7—H7A	120.6	C2—C14—H14B	109.5
C8—C7—H7A	120.6	H14A—C14—H14B	109.5
N2—C8—N1	113.54 (12)	C2—C14—H14C	109.5
N2—C8—C7	123.21 (12)	H14A—C14—H14C	109.5
N1—C8—C7	123.21 (12)	H14B—C14—H14C	109.5
O1—C9—N1	122.02 (13)	H1W1—O1W—H2W1	106.0 (17)
O1—C9—C10	123.12 (12)		
			
C8—N2—C1—N3	−178.82 (11)	C4—C5—C6—C7	178.98 (13)
C8—N2—C1—C5	1.12 (19)	C5—C6—C7—C8	1.0 (2)
C2—N3—C1—N2	178.57 (12)	C1—N2—C8—N1	−179.49 (11)
C2—N3—C1—C5	−1.37 (19)	C1—N2—C8—C7	−1.82 (19)
C1—N3—C2—C3	1.62 (19)	C9—N1—C8—N2	−165.13 (12)
C1—N3—C2—C14	−178.93 (12)	C9—N1—C8—C7	17.2 (2)
N3—C2—C3—C4	−0.5 (2)	C6—C7—C8—N2	0.8 (2)
C14—C2—C3—C4	−179.88 (13)	C6—C7—C8—N1	178.26 (12)
C2—C3—C4—C5	−0.9 (2)	C8—N1—C9—O1	1.8 (2)
N2—C1—C5—C4	−179.98 (12)	C8—N1—C9—C10	−177.97 (12)
N3—C1—C5—C4	−0.04 (18)	O1—C9—C10—C13	117.22 (14)
N2—C1—C5—C6	0.55 (19)	N1—C9—C10—C13	−63.06 (15)
N3—C1—C5—C6	−179.51 (11)	O1—C9—C10—C11	−2.65 (18)
C3—C4—C5—C1	1.12 (19)	N1—C9—C10—C11	177.07 (11)
C3—C4—C5—C6	−179.46 (13)	O1—C9—C10—C12	−121.97 (15)
C1—C5—C6—C7	−1.59 (18)	N1—C9—C10—C12	57.75 (15)

### Hydrogen-bond geometry (Å, °)

**Table:**

*D*—H···*A*	*D*—H	H···*A*	*D*···*A*	*D*—H···*A*
N1—H1N1···O1W	0.833 (18)	2.041 (17)	2.8633 (16)	169.1 (16)
N3—H1N3···Cl1	0.877 (18)	2.213 (18)	3.0870 (11)	175.2 (16)
O1W—H1W1···Cl1^i^	0.891 (19)	2.219 (19)	3.1091 (12)	176.5 (18)
O1W—H2W1···Cl1	0.85 (2)	2.61 (2)	3.3960 (12)	155.3 (16)
C7—H7A···O1	0.93	2.27	2.8298 (17)	118
C11—H11A···O1^ii^	0.96	2.54	3.3742 (18)	145
C13—H13A···O1W	0.96	2.60	3.4997 (18)	157

Symmetry codes: (i) −*x*+1, −*y*, −*z*+2; (ii) −*x*+1/2, *y*−1/2, *z*.

## References

[bb1] Bernstein, J., Davis, R. E., Shimoni, L. & Chamg, N.-L. (1995). *Angew. Chem. Int. Ed. Engl.* **34**, 1555–1573.

[bb2] Bruker (2005). *APEX2*, *SAINT* and *SADABS* Bruker AXS Inc., Madison, Wisconsin, USA.

[bb3] Carmen, A.-R., Garcia-Granda, S., Goswami, S., Mukherjee, R., Dey, S., Claramunt, R. M., Santa Maria, M. D., Rozas, I., Jagerovic, N., Alkorta, I. & Elguero, J. (2004). *New J. Chem. ***28**, 700–705.

[bb4] Goswami, S. & Mukherjee, R. (1997). *Tetrahedron Lett.* **38**, 1619–1621.

[bb5] Goswami, S., Mukherjee, R., Mukherjee, S., Jana, S., Maity, A. C. & Adak, A. K. (2005). *Molecules*, **10**, 929–934. 10.3390/10080929 PMC6147736 18007362

[bb6] Sheldrick, G. M. (2008). *Acta Cryst.* A**64**, 112–122. 10.1107/S0108767307043930 18156677

[bb7] Spek, A. L. (2003). *J. Appl. Cryst.* **36**, 7–13.

[bb8] Yu, M.-M., Li, Z.-X., Wei, L.-H., Wei, D.-H. & Tang, M.-S. (2008). *Org. Lett.* **10**, 5115–5118. 10.1021/ol8018192 18954057

